# Genomic alterations and diagnosis of renal cancer

**DOI:** 10.1007/s00428-023-03700-9

**Published:** 2023-11-24

**Authors:** Xingming Zhang, Hella A. Bolck, Niels J. Rupp, Holger Moch

**Affiliations:** 1 Department of Pathology and Molecular Pathology, University Hospital Zurich, Schmelzbergstr. 12, 8091 Zurich, Switzerland; 2 Department of Urology, Institute of Urology, West China Hospital, Sichuan University, Chengdu, China; 3 Faculty of Medicine, University of Zurich, Zurich, Switzerland

**Keywords:** Genomic alterations, Diagnostic value, Kidney cancer, Renal cell carcinoma, Molecular defined entities

## Abstract

The application of molecular profiling has made substantial impact on the classification of urogenital tumors. Therefore, the 2022 World Health Organization incorporated the concept of molecularly defined renal tumor entities into its classification, including succinate dehydrogenase–deficient renal cell carcinoma (RCC), FH-deficient RCC, TFE3-rearranged RCC, TFEB-altered RCC, ALK-rearranged RCC, ELOC-mutated RCC, and renal medullary RCC, which are characterized by SMARCB1-deficiency. This review aims to provide an overview of the most important molecular alterations in renal cancer, with a specific focus on the diagnostic value of characteristic genomic aberrations, their chromosomal localization, and associations with renal tumor subtypes. It may not yet be the time to completely shift to a molecular RCC classification, but undoubtedly, the application of molecular profiling will enhance the accuracy of renal cancer diagnosis, and ultimately guide personalized treatment strategies for patients.

**Supplementary Information:**

The online version contains supplementary material available at 10.1007/s00428-023-03700-9.

## Introduction

The rapid evolution in renal cancer management highlights the importance of incorporating multiple specialties in decision-making processes, particularly in utilizing novel molecular technologies to enhance personalized diagnosis and treatment approaches [1]. In the past, the classification of kidney cancer has been mainly based on histomorphological characteristics and the corroborating immunohistochemical profile. The increasing knowledge of molecular alterations in renal cancer, coupled with the global adoption of next generation sequencing (NGS), is driving a significant shift in the diagnostic approach from morphology to molecular analysis. Therefore, further stratification and new definition of tumor entities have been proposed [2]. In 2022, the fifth edition of the World Health Organization (WHO) classification of “Urinary and Male Genital Tumours” took these novel developments into account, introducing a classification of renal tumors partly based on molecular features [3]. Such novel molecularly defined epithelial renal tumors include succinate dehydrogenase (SDH)–deficient RCC, FH-deficient RCC, TFE3-rearranged RCC, TFEB-altered RCC, ALK-rearranged RCC, SMARCB1-deficient medullary RCC, and ELOC-mutated RCC. In addition, characteristic gene alterations are recognized in emerging renal tumor entities for which the collection of evidence is ongoing and key features have yet to be defined. These include papillary neoplasms with reverse polarity that are associated with recurrent mutations of *KRAS* [4], biphasic hyalinizing psammomatous RCC that show *NF2* mutations [5], somatic *TSC2*-inactivating mutations that are identified in eosinophilic vacuolated tumors (EVT), and low-grade oncocytic tumors that may be characterized by *MTOR* mutations [6, 7]. *EWSR1::PATZ1* fusions have been recurrently identified in thyroid-like follicular carcinomas [8].

**Fig. 1:**
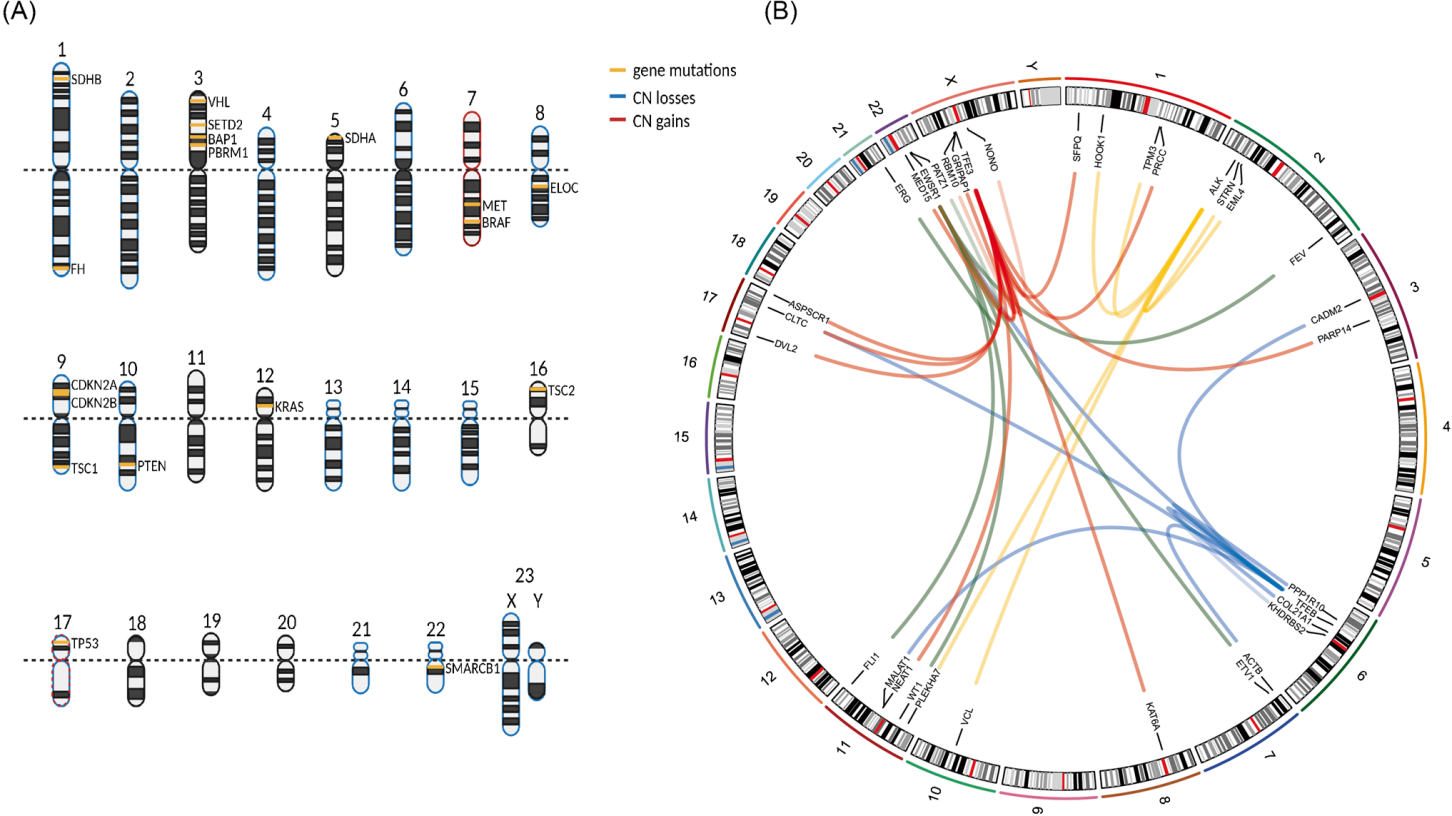
Chromosomal localization of characteristic genetic alterations in various renal cancer subtypes. **A** Genes frequently harboring mutations and common copy number variations. **B** Relevant translocations in renal cancer subtypes

Therefore, the diagnostic workup of rare or unusual renal tumors frequently requires the analysis of complex molecular alterations, including different genetic and genomic alterations. Ideally, the molecular subtyping of renal tumors does not only contribute to the accurate diagnosis, but also provides a basis for personalized treatment. In this review, we discuss the value of specific molecular alterations for the diagnosis of novel and emerging renal tumor types and as a screening tool for hereditary tumor syndromes. As shown in Fig. 1, we outline molecular alterations (mutations, copy number variations, and gene fusions) in renal cancer in the order of chromosomes. We perceive that this will assist pathologists and molecular biologists who interpret molecular tumor analysis or investigate distinct aberrations as part of their translational research. For those, looking for the molecular alterations in a distinct renal cancer entity, we have summarized these in Table 1.

**Table 1:** Gene mutations and SCNAs in renal cancer

Entity	Gene mutations (%)*	SCNAs
ccRCC	VHL (25.5–79.5%), PBRM1 (29.2–54.3), SETD2 (4.1–42.9%), BAP1 (7.1–24.4%), BRAF (2.9%), CDKN2A (1.1%), FH (2.9%), KRAS (0.2%), MET (1.3–5.7%), PTEN (1.9–10.3%), SDHA (0.4–2.9%), SDHB (0.2–0.9%), SMARCB1 (0.9–11.3%), TP53 (2.8–6.4%), TSC1 (0.4–3.1%), TSC2 (0.9–6.4%), ELOC (0.7–4.7%)	Losses of 3p, 1p36; gains of 5q, 8p, 9p, and 14 [9]
chrRCC	TP53 (33%), PBRM1 (1.5%), PTEN (9.1%), SDHA (7.6%), SETD2 (3%), SMARCB1 (1.5%), TSC1 (3%), TSC2 (4.5%), VHL (1.5%)	Losses of 1, 2, 6, 10, 13, 17, 21, and Y [9, 10]
ELOC-mutated RCC	ELOC (100%), BAP1 (9.1%)	
ESC-RCC	TSC2 (71.4%), TSC1 (28.6%), TP53 (14.3%)	
FH-dRCC	FH (96–100%), NF2 (12–16.7%), CDKN2A (1.8%), KRAS (3.5%), MET (5.3%), PBRM1 (8%), PTEN (7%), TP53 (8.8%), TSC1 (3.5%), TSC2 (3.5%), VHL (1.8%)	22q loss [11]
LOT	TSC1 (10%)	
Pediatric Rhabdoid Tumor	SMARCB1 (9.7%)	
papRCC	BAP1 (5%), BRAF (1.4%), CDKN2A (0.7%), FH (0.7%), KRAS (1.8%), MET (7.4%), PBRM1 (3.9%), PTEN (2.5%), SDHA (0.4%), SETD2 (5.7%), SMARCB1 (3.5%), TP53 (2.5%), TSC1 (0.7%), TSC2 (2.1%), VHL (1.1%), ELOC (0.4%)	Gains of chromosomes 7 and 17 [9]
PRNRP	KRAS (44.1%)	
Rhabdoid Cancer	SMARCB1 (2.5%)	
RMC	MET (3.2%), SDHA (6.5%), SETD2 (6.5%), SMARCB1 (6.5%)	Gain of chromosome 8q; loss of chromosome 22 [12]
TC-RCC	MET (23%), TP53 (16%), VHL (17%)	Gain of chromosome 9 and 17 [3, 13]
TFE3-tRCC	FH (1.9%)	
CDC	NF2 (29%), SETD2(24%), SMARCB1 (18%), CDKN2A (12%)	Losses of 1p, 6, 8, 9, 14, and 22 [14, 15]

*ccRCC* clear cell renal cell carcinoma, *chrRCC* chromophobe renal cell carcinoma, *ESC-RCC* eosinophilic solid and cystic renal cell carcinoma, *FH-dRCC* fumarate hydratase–deficient renal cell carcinoma, *LOT* low-grade oncocytic tumor, *papRCC* papillary renal cell carcinoma, *PRNRP* papillary renal neoplasm with reverse polarity, *RMC* renal medullary carcinoma, *TC-RCC* tubulocystic renal cell carcinoma, *TFE3-tRCC* TFE3-translocation renal cell carcinoma, *CDC* collecting duct carcinoma

*Source of the percentages is presented in Supplementary Table 1

## Molecular alterations in the diagnosis of renal cancer

### Chromosome 1

#### Fumarate hydratase

**Fig. 2:**
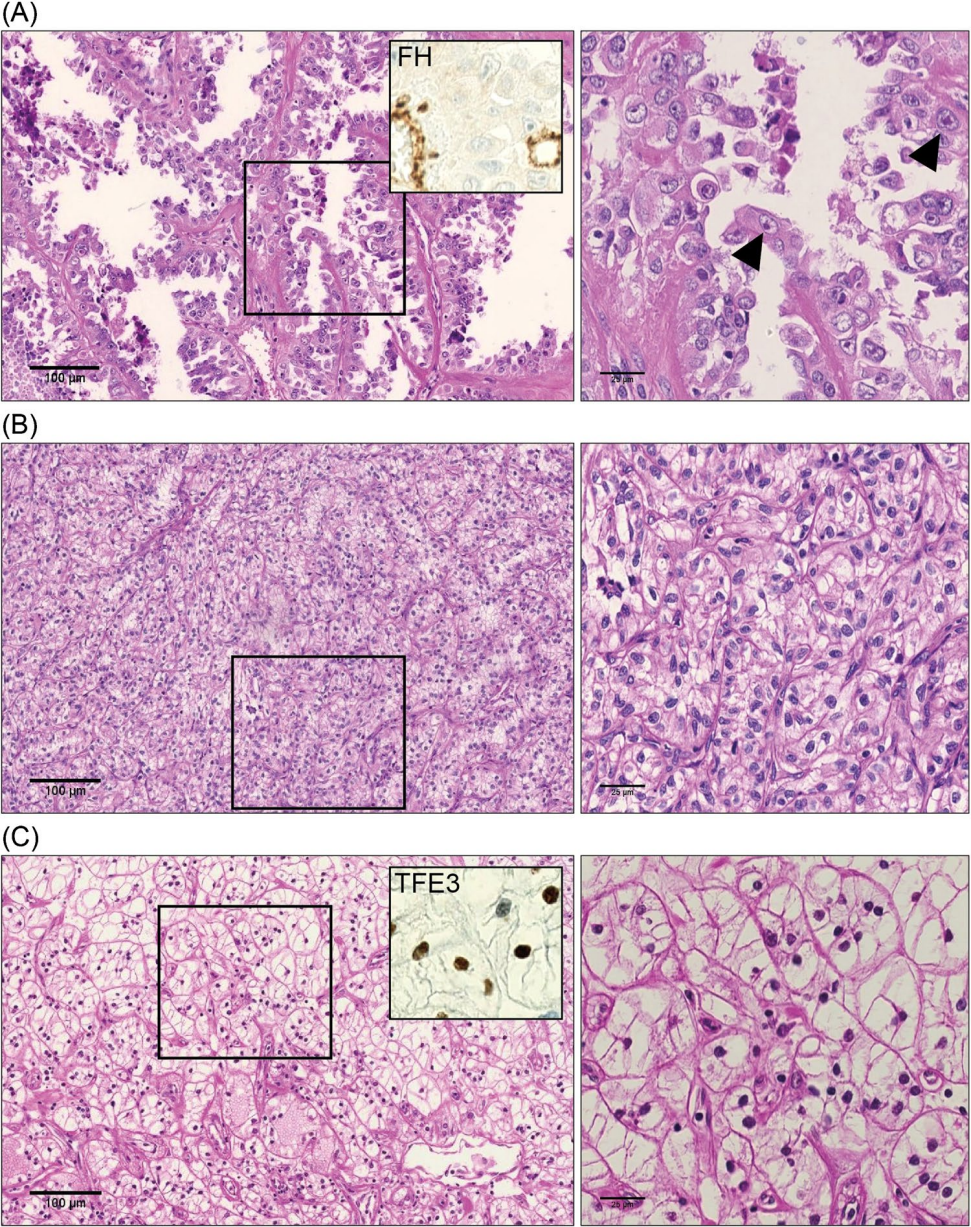
Histology of representative cases of molecularly defined RCC subtypes according to WHO 2022. **A** FH-deficient RCC in which the *FH* mutation p.N154K was detected by NGS analysis (Ref.12). Left panel: H&E staining, with upper-right corner corroborating complete immunohistochemical loss of FH protein expression in the tumor cells (retained in endothelial cells). Scale bar indicates 100 µm; right panel: morphology of the same RCC shown at higher magnification, with prominent nucleoli reminiscent of virus-inclusion bodies (indicated by an arrow), scale bar indicates 25 µm. **B** ELOC-mutated RCC (*ELOC* p.Y79C) discovered through NGS analysis. Left: H&E staining demonstrated clear cell morphology, scale bar indicates 100 µm; right: morphology of the same RCC shown at higher magnification, scale bar indicates 25 µm. **C** TFE3-rearranged RCC with clear cell features in which RNA-based NGS analysis uncovered an *SFPQ::TFE3* fusion. Left: H&E staining demonstrated clear cell morphology, with upper-right inset showing strong nuclear TFE3 immunostaining of the tumor cells. Scale bar indicates 100 µm; Right: morphology of the same RCC shown at higher magnification, scale bar indicates 25 µm

The fumarate hydratase (*FH*) gene, located on chromosome 1q42, encodes for one of the key enzymes involved in the tricarboxylic acid (TCA) cycle. Its main function is to catalyze fumarate into l-malate [16]. Its (bi-allelic) mutation and/or deletion is considered the main molecular event in FH-deficient RCC, formerly classified as hereditary leiomyomatosis and renal cell carcinoma RCC (HLRCC-RCC). Cases presenting with FH germline mutations are often characterized by aggressive RCCs as well as cutaneous and uterine leiomyomas. However, recent evidence suggests that these carcinomas can occur sporadically; thus, in the 2022 WHO classification, FH-deficient RCC includes sporadic and hereditary cases [17]. Notably, widespread use of genetic testing has identified more patients with germline *FH* mutations, suggesting that the prevalence of familial *FH* deficiency may be higher than previously estimated [18]. FH-deficient RCC can show a broad spectrum of morphologies, more commonly depicting papillary and tubulocystic growth pattern with very prominent, viral-inclusion like nucleoli [19]. Figure 2A shows a representative case of the histology of an FH-deficient RCC, which we have published before [19], that requires molecular analysis for diagnosis. In addition, oncocytic (“low-grade”) differentiated RCCs associated with FH-loss have been described [20]. For diagnostic purposes, complete immunohistochemical loss of FH protein expression can be used to identify respective cases [21, 22], but in cases harboring a single nucleotide variant (SNV), FH protein expression might be preserved making genomic testing mandatory in suspicious cases [11, 23].

### Succinate dehydrogenase complex iron sulfur subunit B

Inactivation of succinate dehydrogenase complex iron sulfur subunit B (SDHB) on chromosome 1 [24] leads to the deficiency of the enzyme complex and accumulation of oncometabolites that are also linked to the TCA cycle. This inactivation is associated with SDH-deficient RCC [25]. SDH-deficient RCC usually shows proliferation of bland eosinophilic cells with bubbly cytoplasmic changes and sometimes cytoplasmic inclusions [3]. Importantly, the expression of SDHB is lost in all SDH-deficient neoplasms irrespective of the specific SDH subunit (SDHA, SDHB, SDHC, and SDHD) affected by a genetic mutation. Thus, SDHB immunohistochemistry (IHC) can aid diagnosis [24].

### Copy number alterations of chromosome 1

Losses on chromosomes 1, 2, 6, 10, 13, 17, 21, and Y are common in chromophobe RCC (chrRCC) [26]. Alterations on chromosome 1 also exist in clear cell RCC (ccRCC), collecting duct carcinoma (CDC), nephroblastomas, mucinous tubular and spindle cell RCC (MTSC-RCC), and oncocytomas. Loss of 1p36 can be found in ccRCC indicating worse prognosis [27]. In CDC, losses of 1p, 6, 8, 9, 14, and 22 have been observed [14, 15], which can help to distinguish CDC from other types of RCC and upper tract urothelial carcinoma. Moreover, concurrent loss of chromosomes 1p and 16q indicate a poor prognosis in nephroblastoma and can serve as a rationale for a more intensive chemotherapy [28]. Multiple chromosomal losses involving chromosomes 1, 4, 6, 8, 9, 13, 14, 15, and 22 can be found in MTSC-RCC [12]. Additionally, oncocytomas often show recurrent chromosomal losses in chromosomes 1, 14, 21, X, and Y [12]. In contrast, gain of chromosome 1q is associated with a poor prognosis and has been used as a prognostic marker for nephroblastomas in prospective studies [29, 30].

### Chromosome 2

#### Anaplastic lymphoma kinase

Among the novel renal epithelial tumors included into the 2022 WHO classification, anaplastic lymphoma kinase (*ALK*)-rearranged RCC has been defined as a separate subtype [3]. Chromosomal rearrangements such as those involving the *ALK* gene on chromosome 2p23 can form fusions that produce chimeric proteins. These harbor novel functions and are often both overexpressed and more active than their normal counterparts [31]. The wild-type ALK protein is a receptor tyrosine kinase with strictly confined expression patterns. *ALK* gene fusions lead to chimeric proteins that harbor oncogenic activity.

*ALK*-rearranged RCC appear to be very rare representing less than 1% of all RCC cases but some of the cases described were associated with poor clinical outcomes [32]. Consistently, a diverse set of *ALK* fusion partners have been identified including *VCL*, *TPM3*, *EML4*, *STRN*, and *HOOK1* [32]. Among these, vinculin (*VCL*)::*ALK* gene fusions seem to be distinctive in pediatric patients [33, 34]. Additionally, *ALK::STRN* and *ALK::PLEKHA7* gene fusions have been described in tumors mimicking metanephric adenoma, corroborating the notion that gene fusion partners might impact morphology and even clinical outcomes [32, 35].

Diagnostic testing for *ALK* translocations encompasses primarily fluorescence in situ hybridization (FISH) and NGS. IHC can indicate *ALK* rearrangements displaying strong expression of the fusion protein. However, high ALK protein expression can result from other sources than gene translocation, making molecular testing mandatory [36]. Correctly diagnosing RCC with *ALK* fusions is of high clinical significance as aberrantly active ALK proteins are promising targets for therapy with ALK inhibitors like crizotinib [37].

#### Copy number alterations of chromosome 2

As mentioned for chromosome 1, loss of chromosome 2 is one of the common genetic alteration in chrRCC [26].

### Chromosome 3

#### 3p loss and VHL inactivation

Biallelic inactivation of the *VHL* tumor suppressor encoded on chromosome 3p25-26 is a hallmark of ccRCC. Inactivation occurs by mutation, copy number loss, or promoter hyper-methylation and causes accumulation of *HIF1A* and overexpression of HIF target genes [38, 39]. Due to its prevalence, *VHL* mutations can be used as a corroborating marker in the diagnosis of ccRCC. However, *VHL* mutations have also been described in several other subtypes of renal cancer; for instance, tubulocystic RCC (TC-RCC) (17%) [13], papillary RCC (papRCC) (1.1%) [40], chrRCC (1.5%) [9], and FH-deficient RCC (1.8%) [41]. Taken together, *VHL* mutations are typical (> 80% of ccRCC) [42] but not specific for ccRCC. Moreover, they are largely unrelated to prognostic or predictive parameters thus limiting their diagnostic potential. Most importantly, novel therapies for renal cancer have been developed targeting the VHL-HIF pathway; thus, broad profiling of *VHL* aberrations may open the possibility to administer these drugs to a wide range of patients [43–45].

## PBRM1, SETD2, and BAP1

CcRCCs frequently show simultaneous loss of three other tumor suppressor genes located on chromosome 3p in close proximity to *VHL*: PBRM1 (in about 50% of cases), *SETD2* (in about 20% of cases), and *BAP1* (in about 15% of cases). Like *VHL*, mutations of *PBRM1* tend to occur early in tumor development. Mutations in *PBRM1* and *SETD2* often co-exist while mutations in *PBRM1* and *BAP1* seem mutually exclusive at the clone level, with distinct tumor phenotypes [46, 47]. Recently, it has been shown that multiple subclonal drivers including *PBRM1*, *SETD2*, or *BAP1* mutations contribute to high genetic intra-tumor diversity in ccRCC and impact on clinical outcomes [47]. Albeit still under investigation, it is perceivable that detailed analysis of genetic subclonal architecture may be part of ccRCC diagnosis and influence clinical decision-making in the future.

### Chromosome 5

#### SDHA

*SDHA* is another member of the SDH complex. A gene located on chromosome 5 encodes for it. Similar to *SDHB*, inactivation of *SDHA* causes SDH-deficient RCC. Germline pathogenic variants in the SDHA gene exist but occur in less than 0.3% of the population. As they have a lifetime penetrance of only approximately 1.7%, SDHA mutations identified by large NGS test are generally considered incidental findings unrelated to renal tumors. Importantly, SDHA-deficient RCCs show negativity for both SDHA and SDHB in IHC analysis [24].

#### Copy number alterations of chromosome 5

Studies have reported that structural aberrations in chromosome 5q, 8p, 9p, and 14 may have an impact on the prognosis of ccRCC [48]. Copy number gains in the chromosome 5q region are associated with good prognosis, whereas deletions are associated with adverse effects [49].

### Chromosome 6

#### Transcription factor EB

A gene fusion involving the transcription factor EB (*TFEB*) 6p21 locus was first described in 2001 in a pediatric renal neoplasm [50]. Based on similar morphologies, immunohistochemical profiles and related molecular pathologies *TFEB*-rearranged renal neoplasms were initially grouped together with transcription factor binding to IGHM enhancer 3 (*TFE3*)-rearranged RCCs into the microphthalmia-associated transcription factor (MiT) family translocation carcinoma subtype in the 2016 WHO classification [51]. Besides *TFEB* and *TFE3*, this subfamily of transcription factors includes *TFEC* and *MiTF* [52]. Except for *TFEC*, gene translocations involving all of these factors have been identified in RCC [53]. In the 2022 WHO classification, *TFEB*-altered renal cell carcinomas became a separate entity that also includes RCCs with *TFEB* amplifications [3]. The majority of *TFEB*-translocation RCC have been described in children and young adults [54].

The most frequent 5′ fusion partner of *TFEB* is the *MALAT1* gene on chromosome 11 (t(6;11)(p21;q12) translocation). Interestingly, *MALAT1* encodes for a long noncoding RNA that drives overexpression of the intact *TFEB* protein [55]. Several other fusion partners have recently been described including *KHDRBS2*, *COL21A1*, *CADM2*, *CLTC*, *EWSR1*, and *ACTB* [54, 56].

However, *TFEB*-tRCC is a particularly rare disease that is likely underdiagnosed because it includes a variety of nonspecific morphologies and requires molecular confirmation by RT-PCR, FISH, or RNA sequencing. A *TFEB* break-apart FISH probe can be applied for diagnosing RCCs with *TFEB* translocations. However, RNA sequencing can provide a more efficient approach as it can also detect paracentric inversions that have been described in translocations such as *PPP1R10::TFEB* [56]. These aberrations will yield a false-negative FISH result. Strong nuclear immunoreactivity of the *TFEB* protein can suggest the presence of a *TFEB* fusion or, in very rare cases, also result from *TFEB* amplification. *TFEB*-amplified RCC shows a broad spectrum of morphology and is therefore even more easily misclassified. Importantly, in these cases, *TFEB* amplification occurs without *TFEB* rearrangements. Instead, chromosome 6p amplification including the *TFEB* gene have been described [57, 58]. This raises the possibility to diagnose such cases based on mRNA expression or large-scale NGS that facilitates copy number analysis [59].

### Chromosome 7

#### Mesenchymal epithelial transition gene

*Mesenchymal epithelial transition (MET)* gene is located on human chromosome 7q31 and encodes the MET receptor tyrosine kinase, which acts downstream of the hepatocyte growth factor (HGF). It has important roles in cell proliferation, differentiation, migration, and survival [60]. As a proto-oncogene, mutations in the *MET* gene lead to constitutive activation of the c-Met protein [60]. Often, germline *MET* mutations are observed in the context of hereditary papillary renal carcinoma (HPRCC) [61]. *MET* upregulation is defined as *MET* and/or *HGF* amplification, chromosome 7 copy number gain (the gene locus of both *MET* and *HGF*), and/or *MET* kinase domain mutations. MET upregulation is reported in up to 80% of papRCC [62], whereas *MET* gene alterations are rather rare in sporadic papRCC (< 10%; Table 1) [40]. Consequently, MET inhibitors have shown efficacy in a subset of MET-driven papRCCs [62].

#### BRAF

The *BRAF* gene is located on human chromosome 7q34 and encodes the BRAF tyrosine kinase. The most common *BRAF* mutation is p.V600E, which confers a persistent increase in kinase activity. The mutation triggers abnormal cell proliferation and survival signals that promote tumor development and progression. Frequently, *BRAF* p.V600E mutations have been detected in metanephric adenoma, metanephric adenofibroma, and metanephric stromal tumors [12]. However, despite of this distinct driver mutation, these entities are still morphologically defined tumors.

Notably, in a composite case of metanephric adenofibroma-papillary renal cell carcinoma, both the adenoma and carcinoma components have shown the same *BRAF* p.V600E mutation [63]. In addition, epithelial-dominant nephroblastomas can also harbor *BRAF* p.V600E [64]. Importantly, *BRAF* p.V600E has not been found in clear cell sarcoma of the kidney, congenital mesodermal nephroma, or infantile ossifying renal tumors of infancy. Since it is present in most metanephric stromal tumors, *BRAF* p.V600E detection may support the differential diagnosis of difficult cases [65].

#### Copy number alterations of chromosome 7

PapRCC is frequently characterized by gains of chromosomes 7 and 17. Trisomy of chromosomes 7 and 17 is observed already in small papillary renal tumors suggesting the potential involvement of this amplification in the early stages of tumor development [66]. Notably, gains on chromosomes 7 and mutations or duplications of the *MET* gene have been implicated in synergistically enhance its oncogenic effects [48, 67]. Overall, the presence of chromosomal aberrations involving chromosomes 7 and 17 has emerged as a distinctive feature of papRCC, while the significance of other chromosomal alterations may be less pronounced.

### Chromosome 8

#### Elongin C complex

*ELOC* (formerly *TCEB1*) encodes the elongin C protein, a crucial component of the VHL complex that plays a role in the physiological ubiquitinylation and inactivation of HIF1a. *ELOC* mutation frequently occurs in the VHL-binding site at residue Y79 disrupting the VHL-Elongin C complex and causing Hif1a stabilization and the activation of oncogenic downstream pathways [68]. Importantly, in a recent study, biallelic *ELOC* and *VHL* aberrations were mutually exclusive. Notably, there were no mutations detected in *TSC1*, *TSC2*, or *mTOR* in RCCs with biallelic *ELOC* inactivation [69]. To confirm the diagnosis of ELOC-mutant RCC, proof of *ELOC* mutation is necessary (Fig. 2B).

#### Copy number alterations of chromosome 8

Changes in chromosome 8p may have an impact on the prognosis of ccRCC [48]. Loss of heterozygosity (LOH) in 8p has been correlated with advanced tumor stage, indicating its potential role in tumor development and metastasis [70]. Additionally, loss of chromosome 8 can also exist in MTSC [12].

### Chromosome 9

#### CDKN2A/B

The *CDKN2A/B* gene is located on human chromosome 9p21.3 and encodes three important tumor suppressor proteins, p16INK4a, p14ARF, and p15INK4b. These proteins play key roles in cell cycle regulation and suppression of tumor development. The p16INK4a protein inhibits the activity of CDK4/6 enzymes, prevents cell cycle progression, and inhibits cell proliferation [71]. The mutation, deletion, or hyper-methylation of the *CDKN2A/B* gene will inactivate the function of these inhibitory proteins, thereby promoting the development of tumors [72]. *CDKN2A* alterations can occur in ccRCC, high-grade papRCC, and CDC [12]. *CDKN2A* or *CDKN2B* deletions and other complex genomic abnormalities typically occur in high-grade RCC tumors [73].

### Tuberous sclerosis complex 1

Tuberous sclerosis complex 1 (*TSC1*) gene is located on human chromosome 9q34 and encodes the TSC1 protein. TSC1 is a component of the tuberous sclerosis complex (TSC) and interacts with the TSC2 protein (encoded by the *TSC2* gene, located on chromosome 16p13.3) to jointly regulate the activity of mTOR signaling [74]. Mutations in *TSC1/2* lead to mTOR pathway hyperactivation that drives proliferation and growth of cells that form tumors in the kidney [74].

Biallelic inactivation of *TSC1* or *TSC2* is present in more than 90% of angiomyolipomas. Additionally, *TSC1/2* alterations have been described in novel and emerging renal tumor subtypes including ESC-RCC, eosinophilic vacuolated tumors, TFEB-altered RCC, low-grade oncocytic tumors (LOT), and eosinophilic vacuolated tumors (EVT) [75–78]. Interestingly, tumors exhibiting diffuse CK7 positivity and fibromyomatous stroma may also harbor mutations in the TSC/mTOR pathway, with some cases associated with tuberous sclerosis complex. The debate about whether or not tumors with *TSC* alterations represent a distinct pathologic entity is not fully resolved to date. A significant number of tumors within the RCC “Not otherwise specified (NOS)” category show somatic mutations of *TSC2* or activating mutations of *MTOR* implying that these factors could be distinct tumor drivers [12]. Additionally, *TSC1*/*2* mutations are commonly detected in RCCs characterized by prominent leiomyomatous stroma [12]. Taken together, a broad spectrum of RCC is associated with *TSC1/2* mutations. Hence, the detection of these mutations alone cannot be used to classify renal tumors. However, sequencing of *TSC1*/*2* genes can be significant to corroborate the diagnosis of certain subtypes of RCC (e.g., ESC-RCC) [12].

#### Copy number alterations of chromosome 9

Loss of chromosome 9 has been reported in TC-RCC. LOH events affecting chromosomal regions of 9p have been implicated in unfavorable prognosis and tumor recurrence in ccRCC [79].

### Chromosome 10

#### Phosphatase and tensin homolog gene

The phosphatase and tensin homolog (*PTEN*) gene is located on chromosome 10q23 and encodes a phosphatase that negatively regulates cell proliferation, growth, and survival. Mutations in the *PTEN* gene result in over-activation of the PI3K/AKT/mTOR signaling pathway [80] and are common in different subtypes of RCC, especially in ccRCC and chrRCC [12].

Cowden syndrome, a hereditary multi-system disorder, is characterized by mutations in *PTEN* and pre-disposes patients to RCC, in particular with chromophobe-like morphology [81].

### Chromosome 11

#### Wilms tumor gene 1

The *WT1* gene is located on human chromosome 11p13 and encodes the *WT1* transcription factor that plays a key role in embryonic kidney development. In renal cancer, the *WT1* gene mutation is one of the common genetic alterations and has been reported in several subtypes of renal cancer, including ccRCC and in particular nephroblastoma. Approximately 20% of sporadic nephroblastomas exhibit *WT1* gene mutations [12].

In addition, WAGR syndrome is caused by a germline deletion of chromosome band 11p13, which contains the *WT1* gene. In 45–60% of the cases, patients with WAGR syndrome present with nephroblastoma. Denys-Drash syndrome is linked to a germline *WT1* gene mutation, with a 90% risk of nephroblastoma [12].

### Chromosome 12

#### KRAS

The *KRAS* gene is located on human chromosome 12p12.1 and mutations lead to a sustained increase in the activity of the KRAS protein, causing abnormal cell proliferation and survival signaling [82]. In renal cancer, *KRAS* mutations are rare [83]. Recent evidence suggests they are characteristic for the emerging subtype of papillary renal neoplasm with reversed polarity (PRNRP) [84, 85]; thus, their detection may become relevant for papRCC diagnosis in the future [3].

### Chromosome 17

#### TP53

The *TP53* gene is located on chromosome 17p13.1 and encodes a well-known tumor suppressor that has essential functions in the cellular stress response and genome stability maintenance. Inactivating mutations in *TP53* cause abnormal cell proliferation and tumor formation [86]. In ccRCC, papRCC, chrRCC, and nephroblastomas, *TP53* mutations may be additional tumor drivers, and are associated with tumor progression (“second hit”) [10, 87–89]. Because chemotherapy-induced apoptosis depends on functional p53, *TP53* mutations may be associated with chemotherapy resistance [90].

#### Copy number alterations of chromosome 17

Gains of chromosome 17 frequently occur in papRCC [3, 91] but have been reported also in TC-RCC [3].

### Chromosome 22

#### SMARCB1

The *SMARCB1* (also known as INI1, SNF5, or BAF47) gene is located on human chromosome 22q11.23 and encodes a subunit of the SWI/SNF complex that is involved in the regulation of chromatin structure and gene expression. Consequently, mutations in *SMARCB1* drive aberrant gene expression programs thereby promoting tumor cell proliferation and metastasis [92]. Most commonly, *SMARCB1* inactivation occurs by chromosomal translocations or deletion. Importantly, almost all renal rhabdoid tumors show biallelic loss of *SMARCB1* and thus this is one of the universal features of this tumor type [12]. In addition, *SMARCB1* mutations are found in SMARCB1-deficient renal medullary carcinoma and are accompanied by loss of SMARCB1 protein (INI1) expression on IHC [12]. Moreover, assessment of INI1 loss aids the differential diagnosis between SMARCB1-deficient renal medullary carcinoma and high-grade invasive urothelial carcinoma or collecting duct carcinoma. However, it is necessary to keep in mind, that other RCCs presenting with SMARCB1 deficiency as a secondary event may exist [93, 94].

#### Ewing sarcoma breakpoint region 1 gene

The Ewing sarcoma breakpoint region 1 gene (*EWSR1*) on chromosome 22q12 is rearranged in Ewing sarcomas, an aggressive cancer that can sporadically occur in the kidney [95, 96]. Primary Ewing sarcoma of the kidney are very rare but highly malignant, metastasizing early or recurring quickly. Therefore, it is of crucial importance to distinguish them from other predominantly pediatric renal tumors like Wilms tumor, synovial sarcoma, rhabdomyosarcoma, or clear cell sarcoma of the kidney [97].

Ewing sarcoma of the kidney often present with a small cell histology but for an unequivocal diagnosis, molecular analysis is imperative. Between 80 and 95% of patients harbor a chromosomal translocation between t(11;22) (q24;q12) resulting in a fusion between the N-terminal transactivation domain of *EWSR1* and the C-terminal DNA-binding domain of the *FLI1* gene. The chimeric EWSR1/FLI1 protein acts as a powerful transcriptional activator that promotes cell proliferation and causes genomic instability [98]. Other fusion partners include *WT1*, *ERG*, *ETV1*, *E1AF*, and *FEV*. Importantly, cases with *EWSR1::TFE3* tRCC have recently been reported showing that *EWSR1* gene rearrangements may play a role in MiT family translocation RCC [99, 100]. IHC expression of the protein most common fusion partner FLI1 may suggest the presence of a EWSR1-rearranged Ewing sarcoma of the kidney in about 60% of the cases, but is insufficient for diagnosis [97]. EWSR1 translocations can be detected directly by FISH or RT-PCR. However, as these routine methods can only identify a limited number of fusion partners, are low-throughput and labor-intensive, they are increasingly replaced by NGS-based techniques that are robust, are more sensitive, and require no previous knowledge of the fusion partner.

In addition, *EWSR1* gene fusions partnering with *PATZ1* have been recurrently identified in thyroid-like follicular renal cell carcinoma (TFRCC), which was considered a provisional entity in the 2016 WHO classification [51]. The name results from the follicular arrangement of tubular cells with colloid-like that are reminiscent of thyroid follicles. In general, these tumors are of low-grade and show an indolent biological behavior [8]. However, recently, a case of TFRCC with sarcomatoid differentiation and aggressive behavior has been documented, also harboring the *EWSR1::PATZ1* gene fusion [101].

### Chromosome X

#### Transcription factor E3

The *transcription factor E3* (*TFE3*) gene resides on the Xp11.2 gene locus and the associated protein belongs to the MiT-subfamily of transcription factors. Translocations involving TFE3 are the characteristic event in TFE3-rearranged RCC that has first been recognized in the 2004 WHO classification [102]. *TFE3* is rearranged in around 1–4% of adult RCCs but as it is more prevalent in RCCs of children. It is a rare but often aggressive disease [103]. *TFE3*-rearranged RCCs exhibit a wide spectrum of morphologies making it challenging to diagnose based on histological criteria alone (Fig. 2C). Due to this reason, *TFE3*-rearranged RCC may be particularly under-recognized among older (> 45 years) patients.

Many fusion partners have been described for *TFE3*-rearranged RCCs [104, 105]. As the exact breakpoint site in *TFE3* fusions is usually in-frame, pre-mRNA splicing generates a chimeric mRNA transcript fused at exon–exon junctions [104]. These transcripts encode the N-terminal portion of the fusion partner linked to a range of C-terminal encoding exons of *TFE3*. The three most common translocations include t(X;1)(p11.2;q21), fusing the *PRCC* and *TFE3* genes; t(X;17)(p11.2;q25), fusing the *ASPSCR1* and *TFE3* genes; and t(X;1)(p11.2;p34), fusing the *SFPQ* and *TFE3* genes. Leveraging RNAseq technologies, many more fusion partners have been recently identified, including *NONO*, *RBM10*, *DVL2*, *PARP14*, *GRIPAP1*, *MED15*, *KATA6A*, *NEAT1*, *EWSR1*, and *CLTC* [53, 105]. TFE3 fusion partners often involve genes related to RNA splicing and processing, suggesting their potential role in TFE3-rearranged RCC tumorigenesis. These fusions can activate TFE3 continuously or affect its nuclear localization, driving its oncogenic activity [104, 106]. Nevertheless, the variety of known *TFE3* gene fusions is considerable and likely contributes to the high degree of heterogeneity of *TFE3-rearranged RCC*, both morphologically and clinically. Moreover, the prognosis of *TFE3-rearranged RCC* has been shown to depend on the *TFE3* fusion partner highlighting the importance of its accurate molecular detection [104]. Currently, there is no standardized diagnostic work-up for *TFE3-rearranged RCC* and *TFE3* IHC often yields unreliable results [107]. FISH using break-apart probes for *TFE3* has been the gold standard for diagnosis but similar to *TFEB*-tRCC, small intrachromosomal gene inversions such as *RBM10::TFE3*, *GRIPAP1::TFE3*, *RBMX::TFE3*, and *NONO::TFE3* are impossible to detect by this test [53, 108]. NGS-based technologies that can identify gene fusion events in a partner-agnostic manner have been shown to enable accurate molecular diagnosis of *TFE3-rearranged RCC* and may be even more broadly adopted in the diagnostic routine in the future [59].

## Conclusion

Molecular alterations are increasingly used for classification of renal cancers, particularly in challenging cases involving small biopsies, atypical high-grade tumors, and metastatic tumors with unknown origins. However, these alterations are often not exclusive to one type of renal cancer and unequivocal diagnostics may require the analysis of mutations, copy number aberrations, and translocations with specifically designed NGS panels. The emerging field of precision medicine prioritizes the alignment of patients and treatments based on their genomic characteristics. While the detection of *VHL* mutations alone has neither diagnostic nor prognostic significance, recent studies have shown that 49% of patients with *VHL*-associated RCC have achieved a substantial response to treatment with Belzutifan, a novel HIF-2α inhibitor [43–45]. This suggest that detection of molecular alterations in the VHL/HIF axis could have predictive potential and may be considered in the future to guide treatment decisions.

As sequencing technologies evolve and our knowledge about molecular markers advances, genetic and genomic testing becomes more and more important enhancing the precise classification of renal cancers and aid clinical decision-making. However, correlation with morphological features is mandatory for a comprehensive diagnosis.

### Supplementary Information

Below is the link to the electronic supplementary material.

Supplementary file1 (XLSX 15 KB)

## Declarations

The writing of this review article was in full compliance with ethical standards.
